# Rare-Earth Pretreatment Improves Performance of Reactive Dye Argazol Navy Blue on Banana-Fiber Fabric

**DOI:** 10.3390/molecules30010176

**Published:** 2025-01-04

**Authors:** Ao Du, Yongjie Zheng, Wenqi Jiang, Jie Liu

**Affiliations:** 1 College of Chemistry and Chemical Engineering, Qiqihar University, Qiqihar 161006, China; 15765448495@163.com; 2 Engineering Research Center for Hemp and Product in Cold Region of Ministry of Education, Qiqihar University, Qiqihar 161006, China; 3 School of Light Industry and Textile, Qiqihar University, Qiqihar 161006, China; jiangwenqi0127@163.com

**Keywords:** banana fiber, rare earth, reactive dyes, dyeing

## Abstract

At present, the use of conventional reactive dyes on banana-fiber fabric leads to the problem of excessive salt consumption, which is not conducive to environmental protection. In this experimental study, rare-earth-pretreated banana-fiber fabric was dyed with the reactive dye Argazol Navy Blue. The rare-earth pretreatment was carried out to reduce the level of salt consumption, improve dyeing and fixation rates, and reduce the treatment burden of printing and dyeing wastewater. Dye uptake and fixation rates were used as indicators. Single-change factors were assessed by analyzing different amounts of rare earth, sodium carbonate, and sodium chloride, as well as different fixation times and temperatures, so that the effects of the dye additive on the dyed banana-fiber fabric could be investigated. After appropriate ranges were determined for single-change factors, an orthogonal experiment was carried out to establish optimal parameters for the process of dyeing rare-earth-pretreated banana-fiber fabric with Argazol Navy Blue. These parameters were as follows: the required amount of rare earth was 0.40% (o.w.f.); the amount of dye was 2% (o.w.f.); the amount of sodium chloride was 15 g/L; the amount of sodium carbonate was 9 g/L; the fixing temperature was 75 °C; and the fixing time was 45 min. These optimized process parameters were then used to dye the banana-fiber fabric as a whole. Our results showed that the dye uptake and fixation rates for rare-earth-pretreated banana-fiber fabric dyed with navy-blue dye reached 56.85% and 45.32%, respectively. Finally, the effect of rare-earth pretreatment on dyeing performance was analyzed using FT-IR, SEM, and EDS.

## 1. Introduction

The banana tree is a monocotyledonous plant of the Musaceae family. This plant does not have a real trunk, and its pseudostem is composed of different closely connected leaves [1]. Large numbers of discarded pseudostems and leaves accumulate around banana plants. If this agricultural waste cannot be recycled or reused, it causes environmental pollution and an unnecessary waste of natural resources [2,3]. To address this problem, researchers have studied pseudostems and other waste products of banana plants; it has been found that these products contain large numbers of high-quality plant fibers, along with quantities of pectin. Banana fiber can be obtained from the laminated leaves that make up banana pseudostems [4,5]. Banana fiber can be extracted from pseudostems using mechanical, chemical, and microbial methods. At present, the mechanical method is considered to be the most efficient means of extraction [6,7]. Banana fiber is a type of bast fiber that has good mechanical properties. It has a waist-round cross-section and is hollow. Longitudinally, the horizontal section has a slight bulge. The fiber surface is smooth, and some flat-shaped fibers have holes and cracks [8,9,10]. The morphology of banana fiber is similar to that of ramie fiber and cotton fiber; the properties of ramie fiber and cotton fiber are, therefore, similar to those of banana fiber [11]. The diameter of banana fiber is smaller than that of hemp fiber. Banana fiber is also characterized by low elongation and high strength, along with good inherent tensile properties [12,13]. Applications of banana fiber include sanitary napkins, food products, natural-fiber-based composites, pulp and paper, and textiles. In applications such as these, banana fiber is favored due to its excellent moisture absorption and release properties, its dyeability, and its low birefringence [7,14,15,16].

In the clothing industry, consumers seek both beauty and practicality. Dyeing treatments can add a variety of colors to fabrics and improve their aesthetic appearance [17]. Balakrishnan used an exhaust method to dye banana fiber and cotton fiber with reactive dyes. A characterization test demonstrated that banana fiber exhibited better luster, color development, and washing fastness compared with cotton fiber [18]. As a cellulose fiber, banana fiber is composed of polysaccharide compounds. The secondary hydroxyl groups at the C2 and C3 positions and the primary hydroxyl groups at the C6 position in the molecule can undergo nucleophilic reactions with dye molecules [19]. Therefore, low-cost reactive dyes with strong coloring abilities are used for dyeing banana-fiber fabrics [20]. In the traditional reactive dyeing process, a large amount of salt is often used as a dyeing promoter; this inorganic salt is then discharged into wastewater, increasing the treatment burden of printing and dyeing [21,22].

In the dyeing process, rare-earth products such as mordants can promote the formation of coordination compounds in natural dyes, rare earths, and fibers, thereby improving the color fastness of fabrics dyed with natural extracts, as noted in one study of natural dyes such as brazilin [23]. In addition, the use of rare-earth products is environmentally friendly because it effectively reduces the use of dyeing auxiliaries and the environmental pollution caused by dyeing wastewater. In this study, we investigated the process of dyeing rare-earth-pretreated banana-fiber fabrics with a reactive navy-blue dye. Rare-earth pretreatment was found to have the effect of a dyeing promoter. In brief, the use of rare-earth-pretreated fabrics can reduce the excessive salt usage associated with the conventional dyeing process and deliver improved dyeing performance.

## 2. Materials and Methods

### 2.1. Experimental Materials

The following experimental materials were used in the present work: banana-fiber fabric (Haikou Experimental Station, Chinese Academy of Tropical Agricultural Sciences, Haikou, China); hydrogen peroxide (≥30%, Tianjin Damao Chemical Reagent Factory, Tianjin, China); sodium hydroxide (analytically pure, Liaoning Quanrui Reagent Co., Ltd., Jinzhou, China); refining agent DM-1335; stabilizer DM-1403 (technical grade, Guangdong Demei Fine Chemical Co., Ltd., Foshan, China); sodium pyrophosphate (analytically pure, Shenyang No. 3 Reagent Factory, Shenyang, China); rare earth (99.999%, China Rare Earth Network); Argazol Navy Blue ENW (technical grade, Shanghai Yayun Textile and Chemical Co., Ltd., Shanghai, China); sodium carbonate (≥99.5%, Tianjin Kaitong Chemical Reagent Co., Ltd., Tianjin, China); sodium chloride (≥99.3%, Tianjin Kaitong Chemical Reagent Co., Ltd.); citric acid, penetrant JFC (analytically pure, Tianjin Kaitong Chemical Reagent Co., Ltd.); hydrochloric acid (36–38%, Tianjin Kaitong Chemical Reagent Co., Ltd.); and soap powder (industrial product, Guangzhou Yixin Daily Chemical Co., Ltd., Guangzhou, China).

### 2.2. Laboratory Equipment

The following laboratory equipment was used in our experiments: DK-98-1 electronic thermostatic water bath (Beijing Yongguangming Medical Instrument Factory, Beijing, China); 202-AOS electrothermal constant-temperature drying oven (Tianjin North China Experimental Instrument Co., Ltd., Tianjin, China); 723 Visible Spectrophotometer (Shanghai Spectrometer Instrument Co., Ltd., Shanghai, China); solar climate test machine YG (B) 611-6; wash color-fastness test machine SW-12AC (Wenzhou Darong Textile Standard Instrument Factory, Wenzhou, China); friction-fastness test machine 5716 (Wenzhou Textile Instrument Factory, Wenzhou, China); ZB-A Colorimeter (Hangzhou Zhibang Automation Technology Co., Ltd., Hangzhou, China); Spectrum100 Infrared Spectrometer (Perkin Elmer, Waltham, MA, USA); and scanning electron microscope S-3400 (Hitachi, Tokyo, Japan).

### 2.3. Pretreatment Process

The banana-fiber fabric was cut into several 5 cm × 20 cm strips, and the original fabric weight was recorded. The fabric was dried to constant weight for 3 h in a drying oven and then weighed again. A one-bath desizing–scouring–bleaching method was used for pretreatment. Parameters were set as follows: bath ratio, 1:30; refining agent DM-1335 2 g/L; penetrant JFC (an eco-friendly surfactant with excellent biodegradability and a production process that follows the principles of green chemistry), 2 g/L; stabilizer DM-1403 5.5 g/L; NaOH 4.5 g/L; H_2_O_2_ 12 g/L. Bleaching was carried out at 75 °C for 60 min. The bleach and fabric were gently stirred at intervals. After pretreatment, the fabric was washed in water for 2 min at 80 °C, at 50 °C, and at room temperature, then dried and dyed.

### 2.4. Rare-Earth Pretreatment

Rare-earth ions cause banana fibers to fully expand, which is helpful for the reaction between the hydroxyl groups in the amorphous and partially crystalline regions of the fiber and the rare earth. For this reason, it is better to apply rare earth to banana fiber as a pretreatment before dyeing. In the present work, the rare earth was dissolved in hydrochloric acid and then complexed with aqueous citric acid. The pH was adjusted to make the solution increasingly alkaline until there was no rare-earth precipitation in the solution. The fabric was then pretreated in the rare-earth complex solution for 15 min at room temperature and then removed for use.

### 2.5. Dyeing Process

**Figure 1:**
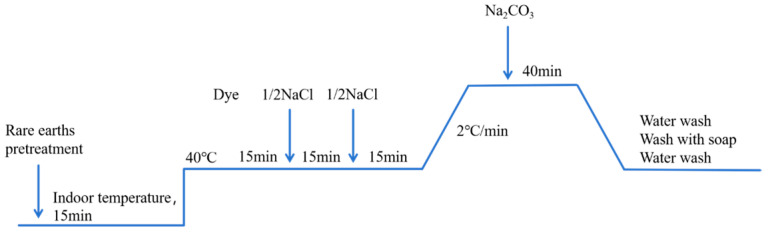
Dyeing process curve.

The dyeing process for rare-earth-pretreated banana-fiber fabric is illustrated in the curve shown in Figure 1.

The dye was prepared with an appropriate amount of distilled water according to a specific bath ratio and then placed in a water bath. A small amount of penetrating agent Penetrant JFC was added to the dye solution. When the dyeing liquid reached a temperature of 40 °C, the rare-earth-pretreated banana-fiber fabric was added, along with half of the NaCl. Dyeing was allowed to continue for 15 min; the remaining NaCl was then added. Next, the temperature was raised to the predetermined dyeing temperature, and Na_2_CO_3_ was added to fix the dye after 15 min. After fixing for a certain time, the water bath was closed to cool the dye solution to room temperature; the banana-fiber fabric was then removed for repeated washing. After washing, the fabric was soaped and boiled at 90 °C for 15 min. After further washing, the fabric was placed in an oven to dry to constant weight. When dried, the fabric was subjected to a series of performance tests.

### 2.6. Percentage of Depletion

Maximum absorption wavelength was determined using a visible spectrophotometer; then, during the fabric dyeing process, 2 mL of dye solution was collected every 15 min, transferred to a 50 mL volumetric flask, and diluted to constant volume. After constant volume was obtained, the absorbance at λmax was measured using a visible light spectrophotometer, and the dye uptake percentage was calculated using Formula (1), as follows:

(1) $$E=Ct\%=\left(1-n*\frac{At}{A0}\right)*100\%$$

where ‘*Ct*%’ is the dye uptake percentage of the dye solution, ‘*n*’ is the concentration multiple of the dye solution and the residual solution at the time of testing, ‘*A*0’ is the absorbance of the dye solution, and ‘*At*’ is the absorbance of the residual solution.

### 2.7. Fixation Rate

After dyeing, the fabric was washed, soaped, rinsed, and dried. The parameters of the soap boiling process were as follows: bath ratio, 1:30; soap powder 2 g/L; Na_2_CO_3_ 2 g/L; soap boiling at 95 °C for 10 min. Two identical portions of soap boiling solution were prepared: one for the soap boiling experiment, and one for the blank control standard soap solution. The standard soap solution was diluted to 250 m; 8 mL was then removed and diluted to 50 mL in a volumetric flask to measure the absorbance C of the standard soap solution. Next, the soap solution at the end of the soaped fabric was also diluted to 250 mL; a 32 mL amount was then removed and diluted in a 50 mL volumetric flask to measure the absorbance D of the residual solution. The fixation rate was calculated according to Formula (2), as follows:

(2) $$F=E-Y\;Y=\frac{D}{C}*N$$

where ‘*E*’ is the dyeing percentage, ‘*Y*’ is the residual amount of the dye that was not dyed after soap boiling, ‘*C*’ is the absorbance of the standard soap solution, ‘*D*’ is the absorbance of the soap solution residue, and ‘*N*’ is the concentration multiple of the soap solution and the residue.

### 2.8. Color Fastness Test

Washing fastness was tested according to GB/T 3921.1-1997 [24] ‘Textiles—Color fastness test—Washing fastness’ standard determination, determination by washing fastness test machine; color fastness to rubbing was tested according to GB/T 3920-2008 [25] ‘Textiles—Color fastness test—Color fastness to rubbing’ standard determination, using a color fastness to rubbing test machine; light fastness was tested according to GB/T 8426-1998 [26] ‘Textile Color Fastness Test Light fastness: Sunlight’ standard determination using a solar climate test machine.

### 2.9. Color Difference Test

Color deviations in the ∆E* values obtained using the CIE Lab color system (L*, a*, b*, c*, and h) were evaluated using a colorimeter to test the banana-fiber fabrics subjected to the two different treatment processes. L* represents the brightness of the object; a* represents the red and green of the object, with positive values representing red and negative values representing green; b* represents the yellow-blue color of the object, with positive values representing yellow and negative values representing blue; c* represents saturation; and h represents the hue angle [27]. Table 1 is a commonly used color-difference comparison table for textiles. From the table, it can be seen that when ∆E is between 0 and 1, the color difference cannot be distinguished by the naked eye. If ∆E is between 1 and 2, the human eye can perceive it slightly; however, if the color sensitivity is not high, it is still invisible. If ∆E is between 2 and 3, the color difference between the substances can be clearly identified. When ∆E reaches 3.5–5, the color difference is very obvious. If ∆E is higher than 5, two colors can be visually identified.

## 3. Discussion

### 3.1. Factors Affecting Dyeing Performance on Banana-Fiber Fabrics

#### 3.1.1. Appropriate Amount of Rare Earth

**Figure 2:**
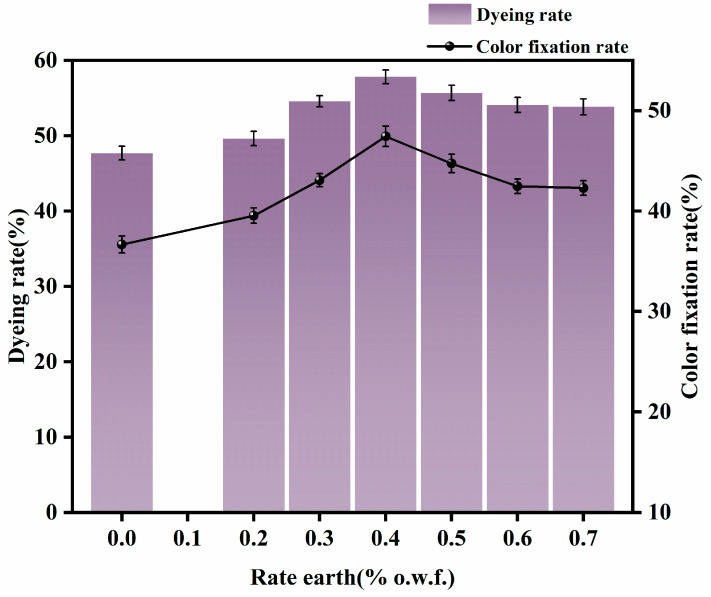
Effects of different rare-earth dosages on dyeing performance.

The banana-fiber fabric was dyed according to the dyeing process shown in Figure 1. The amounts of rare earth were 0% (o.w.f.), 0.2% (o.w.f.), 0.3% (o.w.f.), 0.4% (o.w.f.), 0.5% (o.w.f.), 0.6% (o.w.f.), and 0.7% (o.w.f.). Other parameters were as follows: NaCl 15 g/L, Na_2_CO_3_ 9 g/L, a small amount of Penetrant JFC, dyeing temperature 60 °C, fixation time 40 min. When dyeing was concluded, washing and soaping were carried out, followed by further washing. The dye uptake and fixation rates were then tested. The effects of different rare-earth dosages on dyeing performance are shown in Figure 2.

It can be seen from Figure 2 that when the amount of rare earth rose from 0.2% (o.w.f.) to 0.4% (o.w.f.), the fixation and dye uptake rates both increased rapidly. Because the rare earth and the hydroxyl group on the fiber form a new dye seat, the rare earth and the dye form a reactive-dye–rare-earth-fiber dye complex, the dye combines better with the fiber, the color of the dye solution becomes darker, and the fixing effect is better. With increased amounts of rare earth, however, dye uptake and fixation rates do not continue to increase; instead, they begin to decrease. Because the dye solution is alkaline in the fixing stage, the amount of rare-earth dosage (REE) increases and the unreacted REE ions form a precipitate with the OH in the dye solution, which is not conducive to the diffusion of dye molecules; fixation of the alkali agent to the dye is thus negatively impacted, resulting in poor dye uptake and color fastness in the fabric. Consequently, the appropriate amount of rare earth was determined to be about 0.4% (o.w.f.).

#### 3.1.2. Appropriate Amount of NaCl

**Figure 3:**
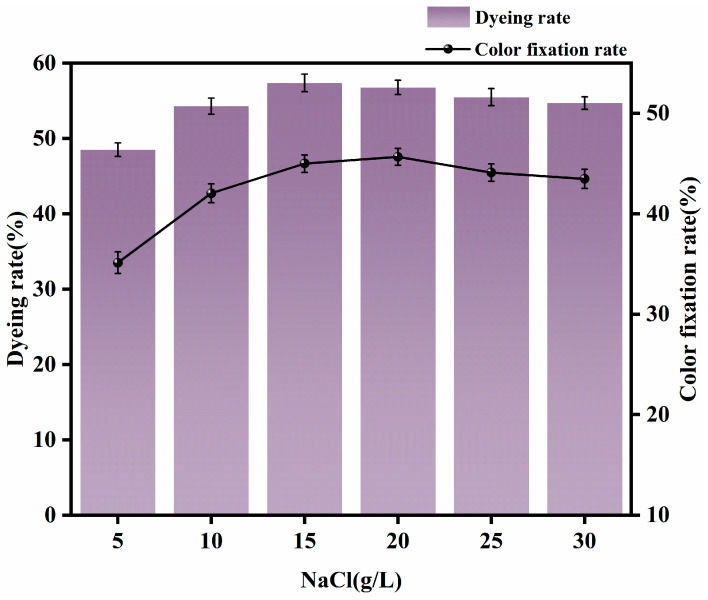
Effects of different NaCl dosages on dyeing performance.

Dyeing was carried out with a rare-earth dosage of 0.4% (o.w.f.), 2% dye (o.w.f.), Na_2_CO_3_ 9 g/L, a small amount of added Penetrant JFC, and NaCl dosages of 5 g/L, 10 g/L, 15 g/L, 20 g/L, 25 g/L, and 30 g/L, with a temperature of 60 °C and a fixation time of 40 min. The fabric was then washed, soaped, and rinsed. Dye uptake and fixation rates were then determined. The effects of different amounts of NaCl on dyeing performance are shown in Figure 3.

It can be seen from Figure 3 that when the amount of NaCl was increased from 5 g/L to 15 g/L, dye uptake increased rapidly, and the fixation rate also increased. Both dyes and fibers are negatively charged. NaCl, as a neutral salt, has the characteristics of small molecules, fast diffusion, and Na ionization. Sodium ions exert an intermolecular electrostatic force, which is adsorbed on the surface of the fiber, so that the negative charge on the surface of the fiber is shielded; as a result, the intermolecular repulsion between the dye and the banana fiber becomes smaller, and more dye molecules enter the fiber, resulting in a better dyeing effect. With increased amounts of NaCl, the rate of improvement in the dyeing rate declines. When the amount of NaCl exceeded 15 g/L, the dyeing rate and fixation rate both showed a downward trend. The reason for this phenomenon is that excessive amounts of NaCl result in excessive accumulation of the dye, causing the reactive dyes adsorbed on the fiber surface to enter a saturated state; this results in precipitation, which not only inhibits dyeing and fixing but also causes drug waste and aggravates the difficulty of wastewater treatment. In light of the information given above, the appropriate amount of NaCl was determined to be approximately 15 g/L.

#### 3.1.3. Appropriate Amount of Na_2_CO_3_

**Figure 4:**
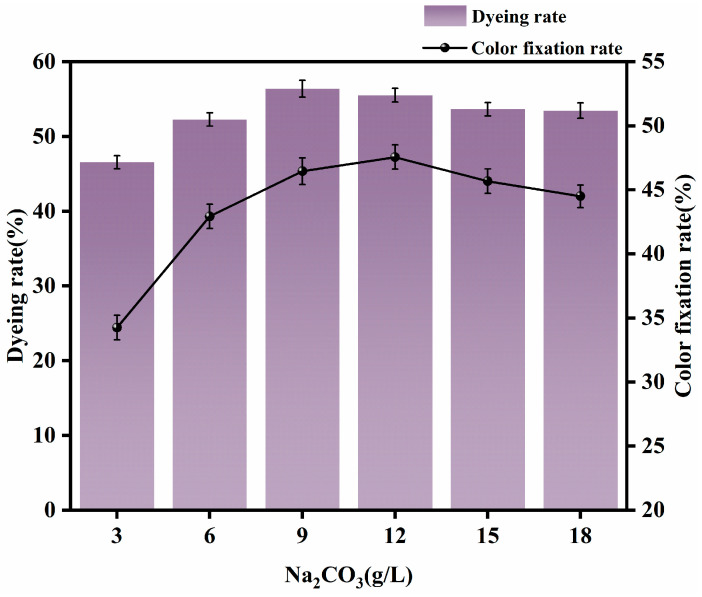
Effects of different Na_2_CO_3_ dosages on dyeing performance.

Dyeing was carried out with a rare-earth dosage 0.4% (o.w.f.), 2% dye (o.w.f.), NaCl 15 g/L, a small amount of Penetrant JFC, fixing at 60 °C for 40 min, and Na_2_CO_3_ dosages of 3 g/L, 6 g/L, 9 g/L, 12 g/L, 15 g/L and18 g/L. The fabric was washed, soaped, and rinsed, then tested for dye uptake and fixation rates. The effects of different amounts of Na_2_CO_3_ on dyeing performance are shown in Figure 4.

From Figure 4, it can be seen that the rate of uptake of Argazol Navy Blue dye on banana fiber increased with increases in Na_2_CO_3_ dosage. When Na_2_CO_3_ was 3 g/L, the fixation rate was low, and the color of the banana fiber was light. With increases in Na_2_CO_3_ dosage, dye uptake and fixation rates both increased rapidly. This is because there is a repulsive force between the dye and the cellulose fiber molecules. When the amount of Na_2_CO_3_ is increased, the alkalinity of the dye solution is increased, the reaction between the dye and the fiber is enhanced, the dye absorption is increased, and the color of the dyed fabric is obviously darker. When the dosage of Na_2_CO_3_ reached about 9 g/L, rates of increase stabilized. Then, when Na_2_CO_3_ dosage was further increased to 12 g/L, the dye uptake rate began to decrease. This is because increasing the dosage of Na_2_CO_3_ above such levels leads to high alkalinity of the dye solution; as a result, the dye hydrolysis rate increases to a level that is higher than the dye absorption rate, the number of dye sites that combine with the fiber decreases, and the alkalinity of the dye solution becomes too strong. The hydroxyl group and the rare earth form a precipitate; this leads to a decrease in the number of dye sites and affects dyeing performance. In addition, the high alkalinity of the dye solution causes the pH value of the wastewater to be excessively high, making it difficult to treat and leading to increased pollution of the environment. In light of the information presented above, the appropriate dosage of Na_2_CO_3_ was determined to be about 9 g/L.

#### 3.1.4. Appropriate Fixing Temperature

**Figure 5:**
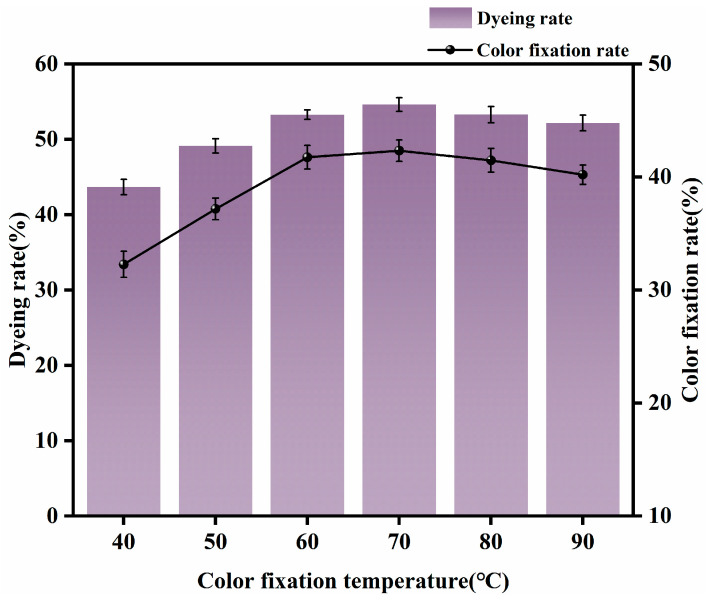
Effects of different fixation temperatures on dyeing performance.

Dyeing was carried out with a rare-earth dosage of 0.4% (o.w.f.), 2% dye (o.w.f.), NaCl 15 g/L, Na_2_CO_3_ 9 g/L, a small amount of Penetrant JFC, fixing time 40 min, and fixing temperatures of 40 °C, 50 °C, 60 °C, 70 °C, 80 °C, and 90 °C. The fabric was washed, soaped, and rinsed, then tested for dye uptake and fixation rates. The effects of different fixing temperatures on dyeing performance are shown in Figure 5.

It can be seen from Figure 5 that when the dyeing temperature was 40 °C, the dye uptake and fixation rates were both low. This is because when the dyeing temperature is low, the diffusion of dye is slow, and the energy barrier that the dye must overcome to diffuse from the dye solution into the fiber is high. With increases in temperature, the dye molecules gain energy, the thermal motion of the dye molecules is intensified, and the diffusion rate is increased. Increased temperature also causes macromolecules in the fiber to enter an active state, and the gap between the fiber molecules increases, thereby increasing the dyeing rate. When the temperature reached 70 °C, there was little or no further improvement with respect to dye uptake or fiber color change. At 80–90 °C, the temperature was already too high. At this level of temperature, the dye is partially hydrolyzed, and dye uptake in the fiber declines as temperature increases further. However, there was little difference between the color of the fiber at these temperatures and the color of the fiber at 70 °C. In light of this, we determined that a dyeing temperature of 70 °C was appropriate, not only because of the good fixing effect obtained at this temperature, but also because of the savings in energy consumption thereby achieved.

#### 3.1.5. Appropriate Fixation Time

**Figure 6:**
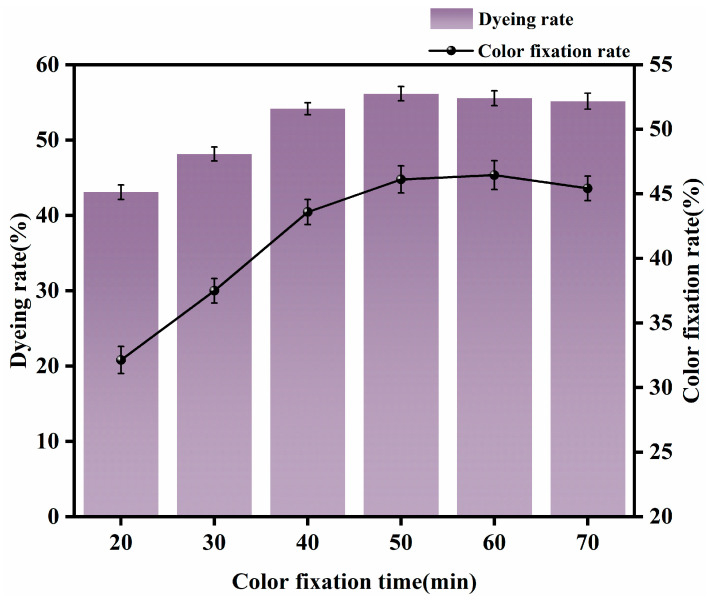
Effects of different fixation times on dyeing performance.

Dyeing was carried out with a rare-earth dosage of 0.4% (o.w.f.), 2% dye (o.w.f.), 15 g/L NaCl, 9 g/L Na_2_CO_3_, a small amount of Penetrant JFC, a fixing temperature of 70 °C, and fixation times of 20 min, 30 min, 40 min, 50 min, 60 min, and 70 min. The effects of different fixation times on dyeing performance are shown in Figure 6.

It can be seen from Figure 6 that when the fixation time increased from 20 to 50 min, dye uptake and fixation rates both increased rapidly, and the color of the fabric gradually darkened. This is because short fixation times result in an incomplete combination of dyes and fibers, and the dyeing effect cannot be further improved. When the fixation time reached 60 min, the dye uptake and fixation rates began to decline because the dye solution during fixation was alkaline. As fixation time increased, the dyes on the fiber were gradually hydrolyzed so that dye that originally adhered to the fabric could no longer do so due to hydrolysis, causing the dye uptake and fixation rates to stop increasing, or even to decrease. In light of the information given above, to maximize the efficiency of the dyeing process and the performance of the fabric, the appropriate fixation time was determined to be approximately 50 min.

#### 3.1.6. Orthogonal Optimization of Dyeing Process for Banana-Fiber Fabric Using Argazol Navy Blue

To assess the impact of single factors, an orthogonal experiment with array L9 (3^4^) was designed to optimize the dyeing process for banana-fiber fabric using the reactive dye Argazol Navy Blue. The experimental results and range analysis are presented in Table 2.

It can be seen from Table 2 that the order of the factors affecting the dyeing rate of Argazol Navy Blue reactive dye is A > C > B > D. The amount of rare earth exerts the greatest influence, and the fixation time exerts the least. The order of the factors affecting the fixation rate of Argazol Navy Blue reactive dye is A > C > D > B. The most influential factor is the amount of rare earth, and the amount of NaCl has the least influence. According to the dyeing effect and the comprehensive cost, the optimal process parameters for Argazol Navy Blue reactive dye were A2B2C3D2; that is, the amount of rare earth was 0.40% (o.w.f.), the amount of NaCl was 15 g/L, the amount of Na_2_CO_3_ was 9 g/L, the fixation temperature was 75 °C, and the fixation time was 45 min. A reproducible dyeing experiment was carried out on banana-fiber fabric using the optimized process, and a performance test was conducted. The dye uptake and fixation rates for banana-fiber fabric dyed with Argazol Navy Blue were found to be 56.85% and 45.32%, respectively.

### 3.2. Comparison of Dyeing Properties of Banana-Fiber Fabric Dyed with Argazol Navy Blue

#### 3.2.1. Color Fastness

Changes in the color fastness of banana-fiber fabrics after two different dyeing processes are shown in Table 3. After testing the color fastness of fabrics dyed using the two processes, it could clearly be seen that the color fastness of the fabrics dyed after rare-earth pretreatment was 0.5~1 degree higher than that of the conventionally dyed fabrics. It was, therefore, proven that pretreatment with rare earth promoted the combination of dye molecules and fibers and that wearability was good.

#### 3.2.2. Chromatic Aberration

**Figure 7:**
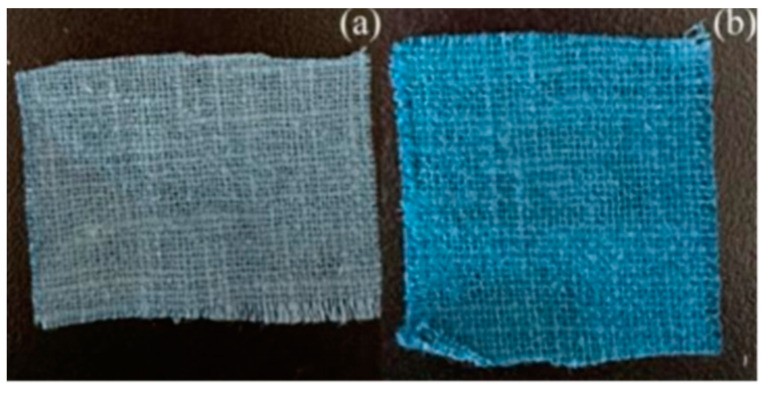
Dyed banana-fiber fabric after (**a**) conventional dyeing and (**b**) rare-earth dyeing.

From the data (Table 4) and visual observations (Figure 7), it can be seen that changes in L* and b* values indicate that the color of banana-fiber fabric after dyeing can be darkened by rare-earth pretreatment; moreover, increases in the c* value prove that the color of the fabric is very light, and that saturation is increased. Rare-earth pretreatment also improves the shade value of the fabric. Therefore, REE pretreatment is essential if deeper and more saturated shades on banana fibers are to be achieved using reactive dyes.

#### 3.2.3. ATR-FTIR Spectroscopy

**Figure 8:**
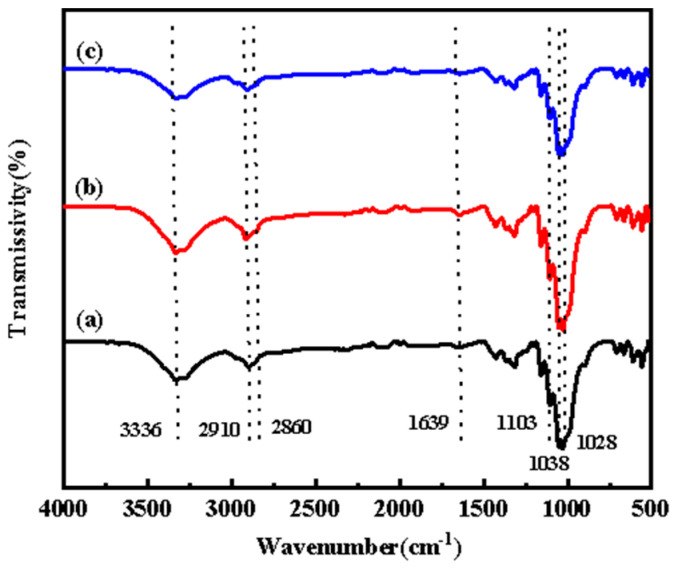
ATR−FTIR spectra of (**a**) banana-fiber fabric after pretreatment; (**b**) banana-fiber fabric after conventional dyeing; and (**c**) rare-earth-pretreated banana-fiber fabric after dyeing.

To assess the combination of reactive dyes and fibers, the Spectrum One Fourier Transform Infrared Spectrometer (PerkinElmer, Waltham, MA, USA) was used to analyze and characterize fibers that were dyed using different dyeing processes. Figure 8 shows infrared spectra for the following: (a) banana-fiber fabric after pretreatment; (b) banana-fiber fabric after conventional dyeing; (c) rare-earth-pretreated banana-fiber fabric after dyeing. In Figure 8a, it can be seen that the stretching vibration absorption band of cellulose and hemicellulose macromolecules O-H in the molecular structure of banana fiber was near 3336 cm^−1^, and 2860–2910 cm^−1^ was the stretching vibration absorption band of saturated C-H (-CH_3_, -CH_2_), both of which are the characteristic absorption peaks of cellulose. The stretching vibration absorption peak at 1639 cm^−1^ represents the skeletal structure of the benzene ring, and is the characteristic absorption peak of lignin in the fiber. In Figure 8b, it can be seen that the characteristic absorption peak of C-O stretching vibration of cellulose macromolecules was in the range of 1103–1106 cm^−1^, and the absorption peak of C-O-C and β-1,4-glycosidic bonds of cellulose and hemicellulose was at 1028 cm^−1^. In Figure 8c, it can be seen that, after rare-earth pretreatment, the C-O stretching vibration characteristic peak at 1038 cm^−1^ changed significantly, indicating that the fiber had a strong combination with the dye and was changed structurally.

#### 3.2.4. Scanning Electron Microscope Analysis

**Figure 9:**
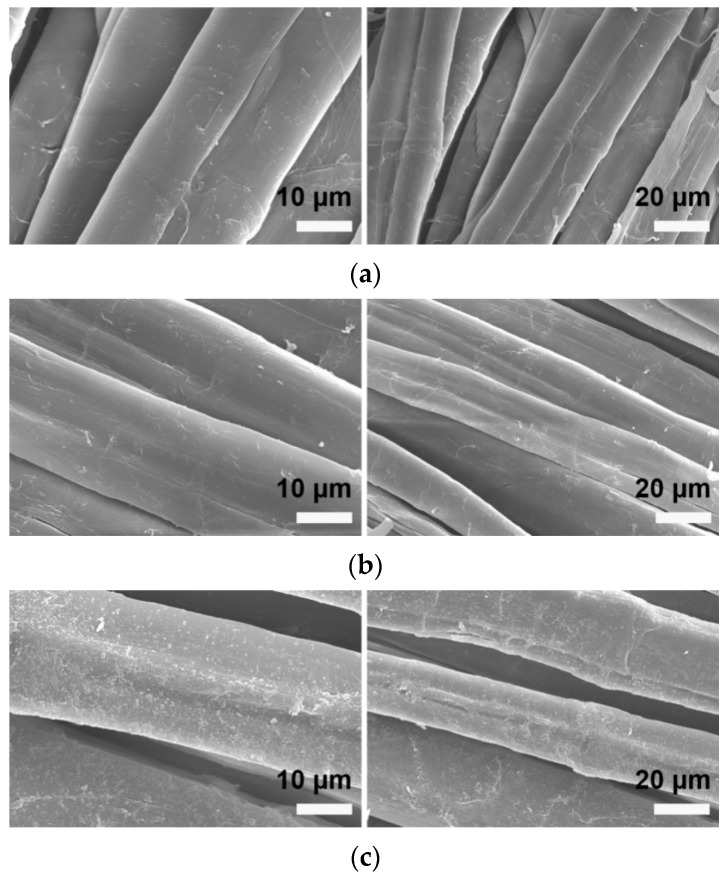
Scanning electron microscope images of (**a**) banana-fiber fabric after pretreatment; (**b**) banana-fiber fabric after conventional dyeing; and (**c**) banana-fiber fabric dyed after rare-earth pretreatment.

To better determine whether there were any changes in the apparent morphology of the banana-fiber fabric before and after dyeing, the fabric was examined using scanning electron microscopy. Figure 9 presents scanning electron microscope images of (a) banana-fiber fabric after pretreatment; (b) banana-fiber fabric after conventional dyeing; and (c) rare-earth-pretreated banana-fiber fabric after dyeing. From Figure 9a, it can be seen that the surface of the pretreated banana-fiber fabric before dyeing is smooth and regular, the thickness of the banana fiber is uneven, the longitudinal direction is straight, the gap between the fibers is close, and the surface of the fiber is damaged but not greatly so. In Figure 9b, it can be seen that, after conventional dyeing, the fiber surface has less etching, and exhibits decreased smoothness and brightness. In Figure 9c, the surface of fabric, which was dried after pretreatment with rare earth, is attached to some granular substances. After dyeing, there are obvious etching stripes on the surface of the fabric, and there are obvious changes in the gaps between fibers. Because rare-earth elements infiltrate the amorphous region of the fabric, promote the swelling of the amorphous region of the fabric, increase the gaps between fibers, loosen the structure, and improve the ability of water molecules to enter the amorphous region, etching streaks are formed on the surface of the fabric. Other impurities on the surface of the banana-fiber fabric are dye particles adsorbed during the dyeing process.

#### 3.2.5. X-Ray Energy Spectrum Analysis (EDS)

**Figure 10:**
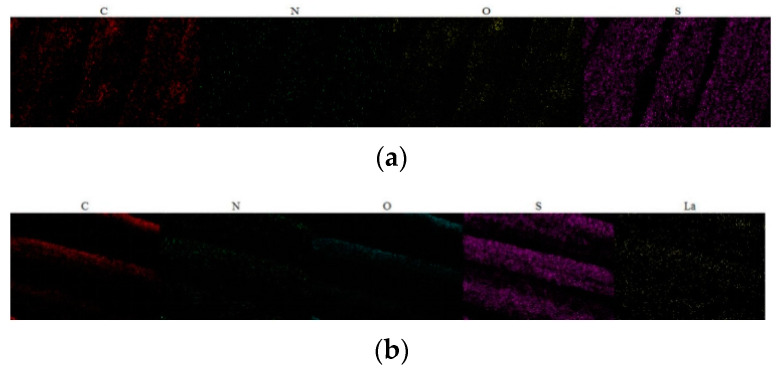
Distribution of surface element content of banana-fiber fabric before and after dyeing using (**a**) conventional dyeing and (**b**) rare-earth-pretreatment dyeing.

EDS was used to analyze changes in elements on the fabric surface. It can be seen in Figure 10a that, after the conventional dyeing process, the surface of the fabric was uniformly dyed with dyes. C, O, N, and S elements can be observed on the surface of the fabric, and N and S elements are expressed as elements in the dye molecule. In addition to C, O, N, and S elements, the rare-earth element La was also detected on rare-earth-pretreated dyed fabric, as shown in Figure 10b. The La element plays a bridging role in the dyeing process to connect fiber and dye molecules, making the dye and fiber more stable. From Figure 10a,b, it can be seen that the content of C, O, N, and S elements in fabric subjected to rare-earth pretreatment prior to dyeing was significantly higher than that in fabric subjected to the conventional dyeing process, proving that rare-earth pre-treatment can improve the effect of the dyes upon fabrics.

## 4. Conclusions

The optimized process for dyeing rare-earth-pretreated banana-fiber fabric with Argazol Navy Blue can be stated as follows: a rare-earth dosage of 0.40% (o.w.f.); a dye dosage of 2% (o.w.f.); a NaCl dosage of 15 g/L; a Na_2_CO_3_ dosage of 9 g/L; a fixing temperature of 75 °C; a fixing time of 45 min; and a bath ratio of 30:1. The performance test results showed that the dye uptake and fixation rates for rare-earth-pretreated banana-fiber fabric dyed with Argazol Navy Blue reached 56.85% and 45.32%, respectively. The dosage of sodium chloride used in this process was much lower than that used in the traditional dyeing process (50 g/L); the objective of low-salt dyeing was therefore achieved. After the performance test and characterization of banana-fiber fabrics, which were dyed using the two dyeing processes, it may be concluded that the color fastness of fabric dyed after rare-earth pretreatment is improved by one level, compared with conventionally dyed fabric. In the present study, we proved that rare earth can be used to improve performance in dyeing banana-fiber fabric with reactive dyes, which may, therefore, be applied as a dyeing aid.

## Figures and Tables

**Table 1:** Color difference comparison table for common textiles.

Chromatic Aberration	Color Difference Size	Color Difference Feeling Degree	Chromatic Aberration	Color Difference Size	Color Difference Feeling Degree
0~0.5	Very small	Trace	3.0~6.0	Large	Identifiability
0.5~1.5	Small	Mild	6.0~12.0	Large	Magnum
1.5~3.0	Smaller	Perceptible	12.0 and up	Very large	Very large

**Table 2:** Orthogonal experimental results and range analysis.

Number	A Rare Earth (%o.w.f.)	B NaCl (g/L)	C Temperature (°C)	D Time (min)	Dyeing Rate (%)	Fixation Rate (%)
1	0.35	10	65	35	47.44	34.25
2	0.35	15	75	45	52.24	38.63
3	0.35	20	85	55	50.20	38.25
4	0.40	10	75	55	52.87	39.49
5	0.40	15	85	35	55.38	44.83
6	0.40	20	65	45	53.43	42.58
7	0.45	10	85	45	53.37	42.85
8	0.45	15	65	55	50.20	40.72
9	0.45	20	75	35	52.74	42.66
K1	49.96	51.23	50.36	51.85		
K2	53.89	52.61	52.61	53.01		
K3	52.11	52.12	52.98	51.09		
R	3.93	1.38	2.63	1.92		
k1	37.04	38.86	39.18	40.58		
k2	42.30	41.40	40.26	41.35		
k3	42.08	41.16	41.97	39.48		
r	5.26	2.53	2.79	1.87		

**Table 3:** Dyeing properties of banana-fiber fabric.

Dyeing Process	Friction Fastness (Grade)		Soap Fastness (Grade)		Fastness to Sunlight (Grade)	
	Trunk	Humidity	Transfer of Color	Discoloration	Transfer of Color	Discoloration
Conventional dyeing	4	3–4	4	3–4	4	4
Dyeing after rare-earth pretreatment	4	4–5	4	4–5	4	4

Notes: Parameters for the conventional dyeing process were as follows: 2% dye (o.w.f.); 50 g/L NaCl; 25 g/L Na_2_CO_3_; fixing temperature of 70 °C; fixing time of 45 min. Parameters for the rare-earth pretreatment dyeing process were as follows: 0.4% rare earth (o.w.f.); 15 g/L NaCl; 9 g/L Na_2_CO_3_; fixing temperature of 75 °C; fixing time of 45 min.

**Table 4:** Color coordinates of banana-fiber fabric.

	L*	a*	b*	c*	h*
Conventional dyeing	43.65	9.78	−18.83	21.22	242.56
Dyeing after rare-earth pretreatment	29.54	32.07	−21.38	38.54	326.31

Notes: Parameters for the conventional dyeing process parameters were as follows: 2% dye (o.w.f.); 50 g/L NaCl; 25 g/L Na_2_CO_3_; fixing temperature of 70 °C; fixing time of 45 min. Parameters for the rare-earth-pretreatment dyeing process were as follows: 0.4% rare earth (o.w.f.); 15 g/L NaCl; 9 g/L Na_2_CO_3_; fixing temperature of 75 °C; fixing time of 45 min.

## Data Availability

Data are contained within the article.
